# The plant microbiome and its importance for plant and human health

**DOI:** 10.3389/fmicb.2014.00491

**Published:** 2014-09-17

**Authors:** Gabriele Berg, Martin Grube, Michael Schloter, Kornelia Smalla

**Affiliations:** ^1^Environmental Biotechnology, Graz University of TechnologyGraz, Austria; ^2^Plant Sciences, University of GrazGraz, Austria; ^3^Environmental Genomics, Helmholtz Zentrum MünchenNeuherberg, Germany; ^4^Julius Kühn-Institut, Federal Research Centre for Cultivated Plants, Institute for Epidemiology and Pathogen DiagnosticsBraunschweig, Germany

**Keywords:** plant microbiome, bacterial communities, endophytes, omics technologies, FISH technology, biocontrol, stress control, plant-microbe interaction

To study plant-associated microorganisms has a long history that reaches back to Lorenz Hiltner's definition of the rhizosphere in 1904 (Hartmann et al., 2008). Today, we know that microorganisms colonizing plant surfaces and inner tissues play an eminent role in shaping of our planet—from our natural vegetation to intense agricultural production systems up to human health. Plant-associated microorganisms have to be considered as key drivers for plant health, productivity, community composition, and ecosystem functioning.

For this e-book “The plant microbiome and its importance for plant and human health” we collected 18 articles, including reviews, original, and opinion articles that highlight the current knowledge regarding plant microbiomes, their specificity, diversity, and function as well as all aspects studying the management of plant microbiomes to improve plant performance and health. The contribution of the single articles of this research topic to these questions is discussed in detail in the mini-review and 1st chapter of the book by Berg et al. (2014a).

Overall the presented articles confirm that the plant-associated microbiome has greatly expanded the metabolic repertoire of plants and often increase resource uptake and provide novel nutritional and defense pathways. Thus, the plant microbiome has a direct impact on plant functional traits, such as leaf longevity, specific leaf area, leaf nutrient levels, and shoot/root ratio. By providing novel nutritional and defense pathways and by modifying biochemical pathways, the plant associated microbiome can enhance or decrease species coexistence and consequently influence not only a single plant but complete ecosystems. Thus, future breeding strategies may take the importance of plant-microbe interactions more into account than in the past, to obtain plants that generate high yields and are more tolerate to the constraints of global change.

Studies related to raw-eaten vegetables are a special show case in this e-book. Here the plant-associated microbiome does not only influence plant performance but strongly contributes to human health. As those microbes are also part of our diet they can either improve human health (Blaser et al., 2013) or cause heavy outbreaks of infectious diseases by transferring possible pathogens (Van Overbeek et al., 2014).

Interestingly, the gathered manuscripts indicate that microbiomes of different environments are not isolated but show interplay. For example, the microbiome of vegetables, humans as well as build environment such as hospitals seems to be well-connected (Berg et al., 2014b). Thus, maintaining microbial diversity in the different environments is an important issue to avoid pathogen outbreaks, which can be often explained by microbial imbalances and poorness (Van Elsas et al., 2012), confirming basic theories of ecology that a loss of native species enhances the probability of invasive species to colonize new environments. Therefore, to maintain and support microbial diversity is of interest to stabilize ecosystems and their resilience toward biotic and abiotic stressors. Biotechnological solutions like probiotics, prebiotics, and synbiotics for plants as well as humans can provide support for the indigenous microbiome (De Vrese and Schrezenmeir, 2008).

The most significant recent advances in plant microbiology involve interdisciplinary approaches that link different methodological approaches including omics-technologies. Due to the new methods available and interdisciplinary research cooperation we have the chance to solve many problems of a changing world, but also to address basic hypotheses and questions of microbial ecology and host microbe interactions. Integrating epigenetics in multi-omics techniques opens existing opportunities for new discoveries (Chen et al., 2014). Therefore, we think this comprehensive e-book especially the many reviews can contribute to hold the current knowledge in our hand. This is a very exciting but also challenging time for all researchers in this field. Major advances will come rapidly!

## Conflict of interest statement

The authors declare that the research was conducted in the absence of any commercial or financial relationships that could be construed as a potential conflict of interest.
